# TransDiscovery: Discovering Biotransformation from Human Microbiota by Integrating Metagenomic and Metabolomic Data

**DOI:** 10.3390/metabo12020119

**Published:** 2022-01-26

**Authors:** Donghui Yan, Liu Cao, Muqing Zhou, Hosein Mohimani

**Affiliations:** Computational Biology Department, School of Computer Science, Carnegie Mellon University, Pittsburgh, PA 15213, USA; donghuiy@andrew.cmu.edu (D.Y.); liuc1@andrew.cmu.edu (L.C.); muqingz@andrew.cmu.edu (M.Z.)

**Keywords:** biotransformation, association network, molecular network, mass spectrometry, metagenomics, microbiome, riboflavin, sutterella, enterobacteria

## Abstract

The human microbiome is a complex community of microorganisms, their enzymes, and the molecules they produce or modify. Recent studies show that imbalances in human microbial ecosystems can cause disease. Our microbiome affects our health through the products of biochemical reactions catalyzed by microbial enzymes (microbial biotransformations). Despite their significance, currently, there are no systematic strategies for identifying these chemical reactions, their substrates and molecular products, and their effects on health and disease. We present TransDiscovery, a computational algorithm that integrates molecular networks (connecting related molecules with similar mass spectra), association networks (connecting co-occurring molecules and microbes) and knowledge bases of microbial enzymes to discover microbial biotransformations, their substrates, and their products. After searching the metabolomics and metagenomics data from the American Gut Project and the Global Foodomic Project, TranDiscovery identified 17 potentially novel biotransformations from the human gut microbiome, along with the corresponding microbial species, substrates, and products.

## 1. Introduction

The human microbiome consists of over 22 million genes [1], many of which are biotransformation enzymes that catalyze various chemical reactions. Currently, there is no systematic approach for assigning a function to these enzymes. Therefore, the activity and substrate specificity of these enzymes and their interactions with the host remain undetermined.

The products of microbial biotransformations could be both health-promoting and detrimental [2]. Health-promoting capacities include digestion of the dietary molecules that are indigestible by human enzymes and promoting absorption of nutrients [3]. These processes provide a great source of energy for the host and prevent the accumulation of toxic molecular byproducts [4,5,6,7]. Detrimental biotransformations include inactivation of drugs by the human microbiome [8]. Moreover, carcinogenic compounds formed during cooking procedures, such as acrylamide, are another example of detrimental biotransformations in the human microbiota [9,10,11,12]. A comprehensive understanding of how microbial enzymes transform dietary and drug molecules is crucial for inferring the effects of the microbiome in health and disease.

Functional profiling approaches are widely used for annotating the function of microbial enzymes [13,14]. However, these techniques suffer from several shortcomings. First, these methods are based on sequence homology, and they fail to correctly assign any metabolic function to ∼80% of microbial enzymes [14,15,16]. Moreover, metabolic function assignments are limited to the superfamily level, and these methods fail to predict the substrate specificity of the enzymes [17,18]. Currently, elucidation of novel biotransformations that microbial enzymes catalyze remains a time-consuming and expensive process that requires isolation of microbial strains and/or purification of microbial enzymes [19], which cannot be performed in a high-throughput manner.

Recent advances in metagenomics have enabled the collection of sequencing data on tens of thousands of human microbial communities as part of the Human Microbiome Project [20,21] and the American Gut Project (AGP) [22]. Moreover, high-throughput mass spectrometry technologies have enabled the collection of tandem mass spectral data on various environments, including the human gut microbiome [22] and human diet [23]. The molecular networking strategy (a network of spectra where similar molecules are connected to each other [24,25]) has further revealed thousands of variants of these known molecules that only appear in the gut environment (absent from food). Some of these variants are the products of the chemical biotransformation of dietary molecules by enzymes from either humans or their microbiome.

In the past, association network methods have been introduced for discovering biotransformations by detecting the cooccurrence of molecular and microbial features across various microbial communities [26,27,28]. In an association network, various statistical tests, including Pearson correlation, Spearman’s correlation, and mutual information, can be used to detect the relationship between a molecular feature and a microbial feature. While Pearson correlation focuses on measuring linear relationships, Spearman’s correlation serves the goal of obtaining monotonic relationships, and mutual information can be used to quantify the mutual dependence between two features. By using different correlation tests, different types of relations between molecular features and microbial features can be discovered.

While association networks have revealed several known and novel microbial natural products and biotransformations, these methods suffer from a common shortcoming that makes their application limited: All correlation-based methods report many strong correlations that do not correspond to any biological interactions, e.g., two features affected by a confounding feature could be correlated without any interaction. Therefore, association networks are dense networks with many spurious edges. These spurious edges require researchers to perform extensive computational searches to identify the true biological interactions from the network.

**Figure 1:**
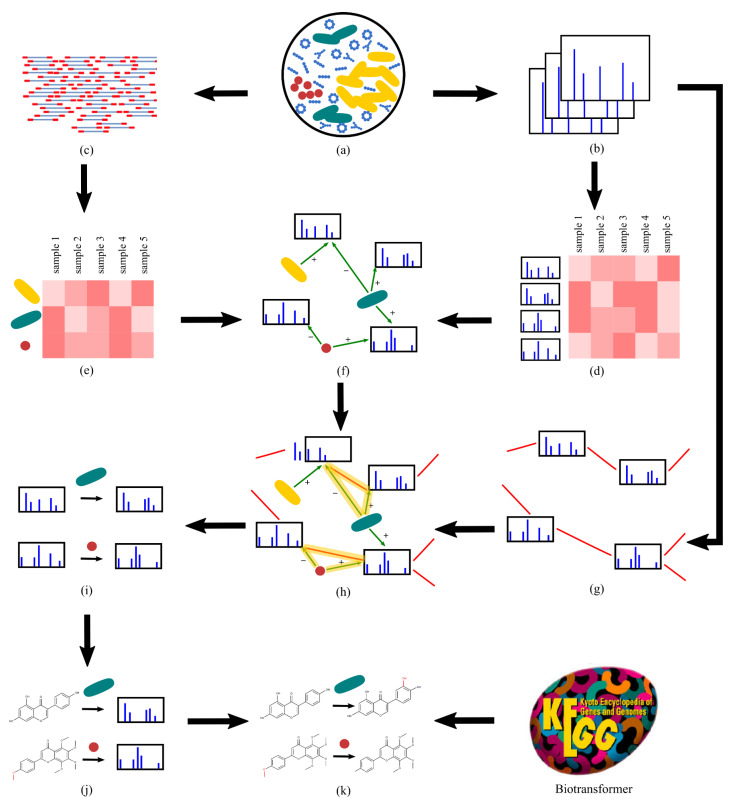
TransDiscovery framework for discovering novel biotransformations of human dietary ingredients by (**a**) the gut microbiome. Starting with (**b**) the mass spectral data of small gut molecules and (**c**) metagenomics data of gut microbes, the pipeline includes the following steps: extracting (**d**) molecular and (**e**) microbial features from raw data, (**f**) constructing an association network [26,27,28] of molecular and microbial features (edges shown in green), (**g**) constructing a molecular network [30] (edges shown in red), (**h**) integrating associations and the molecular network, (**i**) extracting candidate biotransformations as golden triangles, (**j**) identifying substrates of biotransformations with an in silico database search with Dereplicator+ [31], and (**k**) characterizing molecular products of known biotransformations using in silico predictions of BioTransformer [29]. Note that in steps (**f**,**h**–**k**), the nodes can represent either strains or enzymes. In steps (**f**,**h**), the plus and minus labels indicate that the substrate is negatively correlated with the microbial feature and the product is positively correlated with the microbial feature.

In this paper, we develop a culture-independent approach for assigning functions to microbial biotransformation enzymes and specifying their substrates and products based on large metagenomics and tandem mass spectral datasets. We introduce TransDiscovery, a computational framework that integrates molecular networks with association networks and knowledge bases of enzymatic transformations (e.g., BioTransformer [29]) to systematically characterize the microbial biotransformations. TransDiscovery overcomes the challenge of spurious edges in association studies by integrating association networks with molecular networks and biotransformation knowledge bases. TransDiscovery is based on the hypothesis that whenever a microbial enzyme biotransforms a substrate molecule into a product, we observe (i) a strong positive correlation between the enzyme or strain and the product, (ii) a strong negative correlation between the enzyme or strain and the substrate, and (iii) an edge in the molecular network between the substrate and the product (Figure 1). The positive and negative correlations correspond to the increase in abundance of the product and the decrease in the abundance of the substrate during the enzymatic reaction. The edge in the molecular network represents the structural similarity between the substrate and the product. These three relationships are referred to as golden triangles. By finding the triplets of substrates, products, and microbial strains that form a golden triangle, we ensure that the discovered biotransformations are biologically interpretable. With the idea of the golden triangles, the spurious edges will be filtered out from in the network, which decreases the false positives generated by using the correlation approach.

By applying TransDiscovery to the molecular and microbial features in the AGP and Global Foodomics Project (GFoP) datasets, we discovered 17 unique biotransformations of known substrates from PhenolDB [32], along with the corresponding microbial species and molecular products. Our framework is a step forward toward harnessing the big data of genomics/metagenomics, metabolomics, and the existing knowledge bases to illuminate the function of microbial enzymes and their role in health and disease.

## 2. Results

**Overview of TransDiscovery.** The TransDiscovery pipeline (Figure 1) starts with (a) samples of the human microbiome, then collects (b) mass spectra of small-molecule and (c) metagenomics data of microbes and extracts (d) molecular and (e) microbial feature profiles from the data. Afterwards, TransDiscovery constructs (f) the association network [26,27,28] of molecular and microbial features and (g) the molecular network [30] of tandem mass spectra. Further, (h) the integration of these two networks leads to the discovery of (i) candidate biotransformations. The structures of the (j) substrates can be annotated through a database search [31], while (k) the products can be further characterized through the in silico prediction of BioTransformer [29].

**Forming the association network for the AGP dataset.** We constructed an association network for 30,784 molecular features and 11,265 microbial features for 2125 human subjects in the AGP dataset using Spearman’s correlation with a *p*-value threshold of $10^{-4}$ [27]. The molecular features and microbial features were stored in two CSV files. In each file, each row represents one molecular or microbial feature, where each column represents one subject. This resulted in 9,883,612 molecule–microbe associations. Among the obtained associations, 1,379,075 had *p*-values below $10^{-4}$ and absolute rho values over 0.1. Figure S1 shows the frequency of molecular and microbial features in different samples. All the features that formed a significant association appeared in at least a hundred samples.

**Biotransformation of molecules from PhenolDB and HMDB.** We predicted feasible biotransformations for molecules from PhenolDB and HMDB using BioTransformer [29]. The input comprised SMI files that recorded the smile string for each molecule in the database. This resulted in 2364 products for substrates from PhenolDB and 200,833 products for substrates from HMDB.

**Constructing molecular networks.** We constructed a molecular network for the tandem mass spectral data of AGP samples using a cosine threshold of 0.7. The input comprised mzML files that recorded the mass spectrometer output for each sample in the dataset. The resulting network contained 39,219 nodes and 41,296 edges. The molecular network of GFoP had 4950 nodes and 5593 edges. There were 2404 nodes at the intersection of AGP and GFoP, and these nodes had 4809 neighbors that were unique to AGP. Overall, we obtained 45,479 edges by combining the two networks.

**Figure 2:**
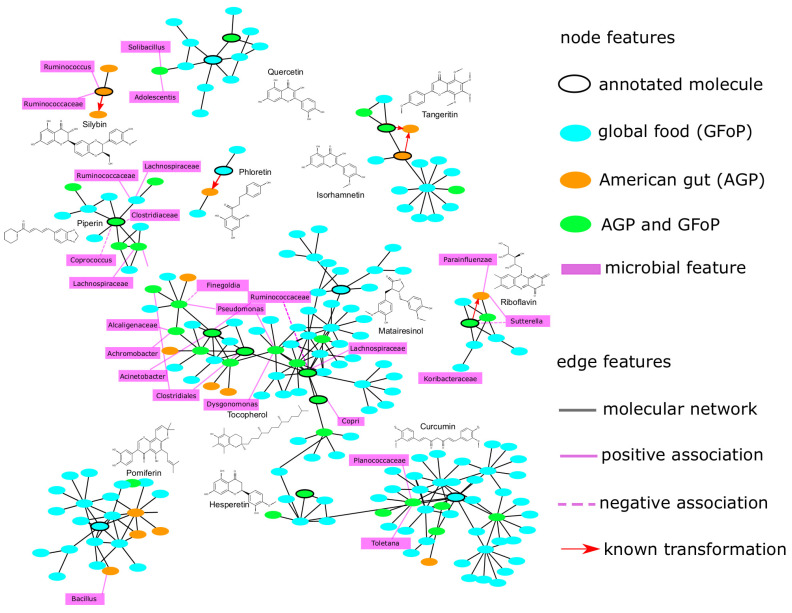
Integrating molecular and association networks. As the size of the network is enormous (41,765 nodes and 45,479 edges), here, we focus on some of the networking families that have a known molecule identified by Dereplicator+ [34] as a polyphenol or a vitamin (25 molecules in total). The network for all 25 molecules is shown in Figure S5. As the association network is currently too dense to visualize, we only show the top two microbial features for each molecular feature (Fisher *p*-value of $10^{-5}$). The known transformations were reported by BioTransformer [29]. By focusing solely on edges in the molecular network and association network, one can get a large number of potential biotransformations with a high chance of being spurious. However, focusing on the overlap between the two networks (golden triangles) results in a much smaller set of potential biotransformations, where many of them can be validated by a literature search.

**Integrating the association network, molecular network, and predicted biotransformations.** We integrated three previously obtained feature pair lists (Figures S2 and S3) to extract more reliable biotransformations (Figure 2). Specific mass annotations for the substrate identification process are listed in Table S1. Specific details of the molecular features in the identified biotransformations are listed in Table S2. Table 1 shows 17 biotransformations from PhenolDB that were retained based on Spearman’s rank correlation coefficients between the substrates or products and the strains. While most of the identified biotransformations held negative/positive correlations between microbial features and substrates/products, we noticed that for the decarboxylation of hydroxycinnamic acids by *Lactobacillus* [33], which has previously been reported in the literature, the same sign was shared in two correlations. The interpretation is that for some biotransformations, a higher abundance of the substrate results in a higher yield of the product.

**Biotransformation identification with enzymatic features.** We applied PICRUSt [35] to 11,265 taxonomy-annotated microbial features to obtain 1535 enzymatic features. These features were treated as the microbial feature input for TransDiscovery. We constructed an association network between these enzymatic features and 30,784 molecular features to obtain 7,075,238 molecule–enzyme associations with Spearman’s correlation *p*-value of less than $10^{-4}$. These associations were integrated with the previously generated molecular network and substrate–product pairs from PhenolDB. The resulting 17 biotransformations are shown in Table S3.

**Biotransformation identification with shotgun sequencing data.** The shotgun sequencing data were available for 145 samples in the AGP dataset. We used KofamKOALA [36] to extract 1074 enzymatic features from the shotgun sequencing data and obtained 4597 associations between these enzymatic features and molecular features with a *p*-value cutoff of $10^{-3}$. However, due to the limited sample size, we failed to identify any significant biotransformations in these association results (Table S4).

**Figure 3:**
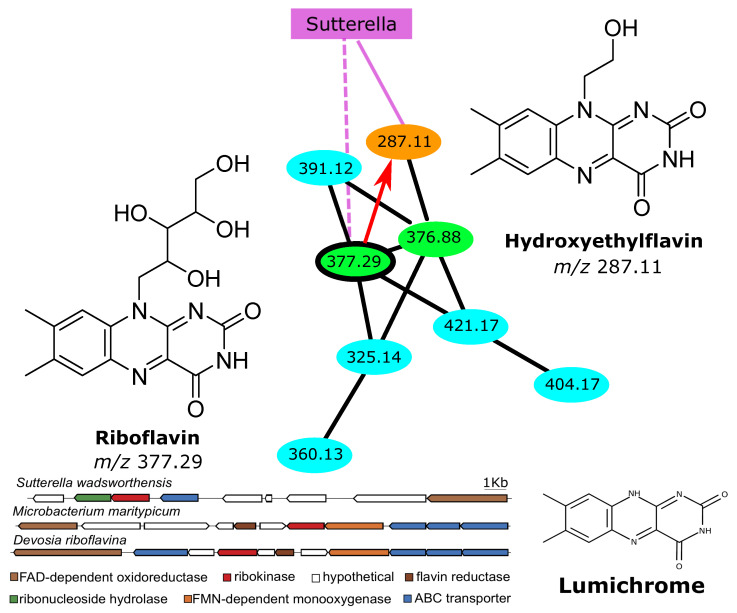
One of the molecular features (orange circle) in the molecular network (same color scheme as in Figure 2) is specific to AGP, and Dereplicator+ [34] identified it as hydroxyethylflavine, a known product of the degradation of riboflavin by an unknown microbial enzyme in gut microbiota [37]. A microbial feature annotated as *Sutterella wadsworthensis* is positively correlated with this product and negatively correlated with riboflavin (highlighted green circle). The predicted riboflavin degradation gene cluster in *Sutterella wadsworthensis* is shown, along with two known riboflavin degradation gene clusters from *Microbacterium maritypicum* and *Devosia riboflavina*. *Sutterella wadsworthensis* is predicted to degrade riboflavin to hydroxyethylflavine, while the other two bacteria degrade it to lumichrome.

**Degradation of riboflavin by*****Sutterella*****.** TransDiscovery identified a variant of riboflavin (Vitamin B2) with *m*/*z* 287.11 that was unique to the AGP dataset. Dereplicator+ [34] identified this variant as hydroxyethylflavine (Figure 3), which is a known product of the degradation of riboflavin by an unknown microbial strain in the human gut microbiota [37]. TransDiscovery identified a microbial strain that was negatively correlated with riboflavin and positively correlated with hydroxyethylflavine (*p*-value threshold of $10^{-5}$). The 16S rRNA of this microbial feature had 99.85% similarity to the *Sutterella wadsworthensis* strain SW4. Further genome annotations revealed that this strain had a gene cluster with ribonucleoside hydrolase and ribokinase, which are known to play a role in the degradation of riboflavin in *Microbacterium maritypicum* [38] and *Devosia riboflavina* [39]. The gene cluster of *Sutterella* is quite different from those of *Microbacterium* and *Devosia*, and its molecular product is also slightly different (hydroxyethylflavine is predicted in the case of *Sutterella*, versus lumichrome in the case of *D. riboflavina* and *M. maritypicum*), suggesting that this riboflavin degradation pathway might be novel.

**Decarboxylation of hydroxycinnamic acids by*****Enterobacteria*****.** The association networks revealed a strong negative correlation between various *Enterobacteria* species and p-coumaric acid (*m*/*z* 165.054), ferulic acid (*m*/*z* 175.064), and caffeic acid (*m*/*z* 181.094). The same *Enterobacteria* species showed strong positive correlations with molecular features with *m*/*z* 121.065, 137.060, and 151.074. These features matched the masses of p-coumaric, caffeic, and ferulic acid after the loss of CO${}_{2}$ (43.989 Da). BioTransformer predicted that p-coumaric, caffeic, and ferulic acid are decarboxylated in the human gut environment. Moreover, decarboxylation of hydroxycinnamic acids by *Enterobacteria* has been previously reported in the literature [40]. Associating molecular features against KEGG enzymes further revealed strong positive associations of molecular features at *m*/*z* 121.065, 137.060, and 151.074 with phenolic acid decarboxylase (K13727), an enzyme frequently observed in *Enterobacteria*, *Lactobacillus*, and *Actinomyces*.

**Dihydroxylation of hydroxybenzoic acid by*****Blautia*****.** In the association network results, a strong negative association between hydroxybenzoic acid (*m*/*z* 139.039) and *Blautia* and a strong positive association between molecular features with *m*/*z* 123.044 and *Blautia* were identified. The mass for this molecular feature matched the loss of an oxygen atom (15.99 Da). It has been previously reported that hydroxybenzoic acid undergoes a dihydroxylation reaction with 4-hydroxybenzyl CoA reductase from the *Blautia* species [41,42].

## 3. Discussion

In this paper, we introduce TransDiscovery, a powerful method for systematically identifying microbial biotransformations by combining a molecular network, association network, and knowledge bases of microbial enzymes. Experimental advances in high-throughput sequencing and mass spectrometry technologies have enabled the collection of genomic and tandem mass spectrometry data from tens of thousands of human microbial communities. Tens of thousands of microbial features (microbial species) and molecular features (molecular substrates and products) from these communities, coupled with increasingly accessible knowledge bases of microbial enzymes, provide great opportunities to dissect the complex biotransformations from the human microbiome. By applying TransDiscovery to the existing database, 17 biotransformations were identified. While some of the biotransformations were validated by a literature search, others are potentially novel biotransformations for further experiments.

The Human Microbiome Project (HMP) has provided a comprehensive catalog of microbial genes in human microbiota. While this catalog is a gold mine for future studies of human health and disease, in order to fully utilize the promise of the HMP, we need to have a better understanding of the mechanisms of action for these genes and how they affect the human host.

Currently, there are no high-throughput technologies for revealing the activity of microbial enzymes, their substrates’ specificity, their molecular products, and their roles in health and disease. Tandem mass spectrometry is a promising technology for high-throughput identification of the substrates and products of microbial biotransformations. However, in contrast to the computational techniques available for high-throughput analysis of metagenomics data of the microbiome, well-established methods for tandem mass spectrometry data analysis are not available.

In the past, association studies have been applied for the detection of molecular products of microbial biotransformations. However, these techniques report many spurious pairs, as they are based on correlation, rather than causation. TransDiscovery improves association networks by integrating them with molecular networks and biotransformation knowledge bases to reliably discover microbial biotransformations.

After searching metagenomics and metabolomics data from the American Gut Project and Global Foodomics Project, TransDiscovery reported 17 potential biotransformations. Several of these biotransformations were validated by a literature search. Currently, there is no comprehensive database of microbial biotransformations. By searching against the most extensive databases, such as HMDB [43] and MetaCyc [44,45], only a few of the substrates identified by TransDiscovery had any reactions assigned to them. Moreover, the enzymes involved in these reactions are usually unknown. The remaining biotransformations predicted by TransDiscovery are potentially novel, and validating them requires further experimental investigations.

Discovery of novel biotransformations is a computationally laborious task, and usually, each paper in this area reports a single novel biotransformation. TransDiscovery enables the discovery of numerous biotransformations in a single study. Validation of the biotransformations predicted by TransDiscovery requires culturing of the predicted microbial strains in media containing the substrate and screening for the presence of the product molecule over time. Validation of the predicted gene clusters further requires knock-out experiments. Wet-lab validation can become a path to the confirmation of the results of TransDiscovery.

While so far, we mainly focused on reporting novel biotransformations identified by combining the AGP and GFoP datasets with PhenolDB, TransDiscovery can take any knowledge base of interest as input. Compared to PhneolDB, other knowledge bases, such as HMDB and FooDB, hold hundreds of times more chemical structures of small molecules from the human body. In the analysis between the AGP and GFoP datasets and HMDB, we were able to obtain hundreds of candidate biotransformations with an even more stringent rho value threshold, which can be used for further experimental validation.

One of the limitations of reliance on marker gene data (16S) for the annotation of microbial features is that it makes it difficult to conduct accurate functional profiling analyses. Currently, TransDiscovery supports the incorporation of shotgun metagenomics data, which enable higher-resolution functional annotation. With shotgun metagenomics data, the identified biotransformations can be linked to the specific enzymes. This will not only help further screen valid biotransformations, but can also provide insight for interpretation and follow-up experiments. However, shotgun metagenomics are more expensive, and currently, there are only a limited number of public samples (a few hundred) with paired shotgun metagenomics and tandem mass spectrometry data. Therefore, it has been impossible to detect any highly significant associations based on shotgun metagenomics data.

Another limitation of the existing approaches is that, while extensive genomics data are available from reference microbial isolates, currently, reference metabolomics datasets from microbial isolates are not available. Reference genomics data have made it possible to map a large number of microbial features to their corresponding taxonomies, but currently, the majority of molecular features of the microbiome remain orphans, as it remains unclear whether they are produced by the host or the microbial strains. While TransDiscovery is a step forward toward annotation of the microbiome metabolites in complex datasets, the availability and incorporation of metabolomics data from reference microbial isolates can vastly increase the power of such computational approaches.

In conclusion, TransDiscovery provides a culture-free approach for assigning functions to microbial enzymes from complex microbial communities that does not require isolation of the microbes and purification of the enzymes. Applications of this strategy include high-throughput characterization of the biotransformation products of dietary molecules by enzymes from the human microbiota. We believe that with the rapid growth of mass spectrometry/metagenomics datasets and knowledge bases, TransDiscovery can become a crucial tool for better understanding gut microbial mechanisms.

## 4. Materials and Methods

**Datasets.** The AGP dataset [22] contains LC-MS/MS and 16S rRNA data from the human gut microbiomes of 2125 human subjects. Shotgun metagenomics data are also available for some of the samples. The GFoP dataset [23,46] contains LC-MS/MS data for 3579 food and 116 beverage samples. When generating features for the association network, Optimus [47] was used to extract 30,784 molecular features from the LC-MS data from the AGP dataset, and QIIME [48] was used to extract 11,265 unique microbial features from the 16S rRNA data based on the Green-Genes Database as the reference. The precursor ion mass tolerance was set to be 0.02 Da, and the retention time tolerance was set to be 5 for Optimus (default parameters). When generating nodes for the molecular network, LC-MS/MS spectra from AGP and GFoP were merged using MSCluster [49]. The precursor ion mass tolerance was set to be 0.02 Da for MSCluster (recommended by MSCluster for qTOF data). The resulting molecular network contained 41,765 nodes. The molecular features from Optimus and the molecular network were combined using a mass tolerance of 0.02 Da. We additionally annotated the extracted molecular features using a spectral library search and Dereplicator+ with default parameters. The spectral library search was level 2 identification, and Dereplicator+ was level 4 identification of metabolites, according to the Chemical Analysis Working Group Metabolomics Standard Initiative [50]. The Phenol Database (PhenolDB) [32] is a public database containing the chemical structures of 370 phenolic compounds. The Human Metabolome Database (HMDB) [43] is a public database containing the chemical structures of 41,919 small molecules from the human body.

**Liquid chromatography mass spectrometry.** The AGP dataset was collected on an UltiMate 3000 UHPLC system equipped with a reverse-phase C18 column coupled to a Bruker Impact HD quadrupole time-of-flight (qTOF) mass spectrometer [22]. The GFoP dataset was collected on an UltiMate 3000 UHPLC system equipped with a reverse-phase C18 column coupled to a Maxis qTOF Impact II mass spectrometer [23].

**Molecular feature extraction.** Molecular features of the LC-MS/MS data were extracted using Optimus [47]. The result was a feature intensity matrix FI, where thecell FI(x,s) represents the intensity of feature *x* in sample *s*. Features present in less than two samples were discarded.

**Microbial feature extraction.** Microbial features with taxonomy annotations used in the main analysis were extracted from the 16S rRNA data using QIIME [48]. The result was OTUMatrix, where the cell OTUMatrix(y,s) represents the count of OTU *y* in sample *s*. The strain annotation listed in Table 1 was obtained by matching the 16S rRNA data with the NCBI RefSeq database using BLAST alignment. The annotation scores are listed in Table S5. Additionally, we used PICRUSt [35] to predict the KEGG enzymes associated with taxonomies based on their 16S rRNA data. This resulted in EnzymeMatrix, where EnzymeMatrix(z,s) represents the abundance of KEGG enzyme *z* in sample *s*. Enzymatic features from shotgun sequencing data were identified using KofamKOALA [36]. This resulted in *Shotgun-EnzymeMatrix*, where *Shotgun-EnzymeMatrix(e,s)* represents the presence or absence of KEGG enzyme *e* in sample *s*. OTUMatrix, EnzymeMatrix, and Shotgun-EnzymeMatrix were correlated with the molecular features.

**Association network construction.** An association network was constructed by calculating a pairwise association test between molecular features and microbial features [27]. The statistical test used was Spearman’s correlation test. Given the molecular feature *x* and microbial feature *y*, the null hypothesis assumed that “the abundance of molecular feature *x* in samples” and “the count of microbial feature *y* in samples” were independent. If the probability of null hypothesis $P_{x,y}$ was lower than a threshold $P_{threshold}$, then the null hypothesis was rejected and (x,y) were reported as associated.

**Molecular network construction.** A molecular network was constructed using the global natural-product social molecular networking (GNPS) infrastructure [30]. First, all of the MS/MS spectra were clustered by MSCluster [49], and identical spectra were merged into the same clusters and represented as nodes in the network. Then, the nodes were matched pairwise using the modification-tolerant spectral matching scheme [24]. Edges in the molecular network were formed when two nodes had cosine scores higher than a threshold of 0.7. The cosine score threshold of 0.7 was recommended by the Molecular Network software as the default value.

**Figure 4:**
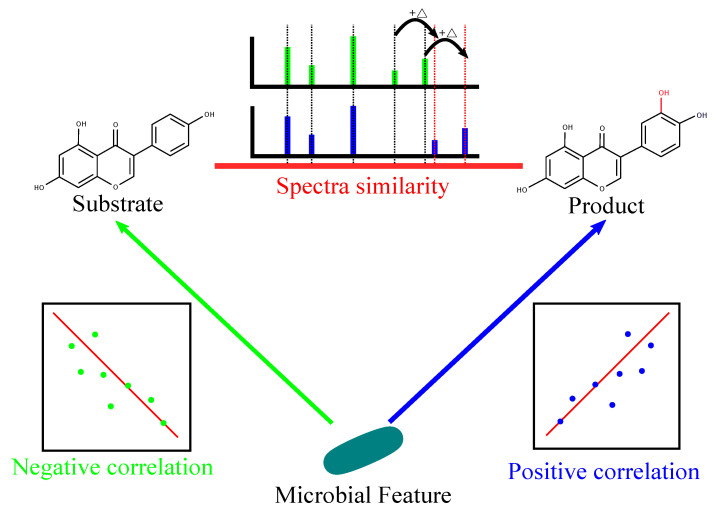
Candidate biotransformations were identified as triplets of microbial features, substrates, and products where there was (i) a positive correlation between the microbial feature and the product, (ii) a negative correlation between the microbial feature and the substrate, and (iii) an edge in the molecular network between the substrate and the product.

**Identifying candidate biotransformations.** In the molecular network, if two spectra (nodes) were very similar (high cosine similarity score), they were connected with an edge. Generally, spectral similarity implies structural similarity [51]. Under the hypothesis that the substrate and product of a biotransformation are structurally similar, candidate biotransformations were identified as triplets of microbial features, substrates, and products where there was (i) a positive correlation between the microbial feature and the product, (ii) a negative correlation between the microbial feature and the substrate, and (iii) an edge in the molecular network between the substrate and the product (Figure 4).

**Substrate identification.** The substrate molecular features were identified by searching them against a chemical structure database (e.g., PhenolDB [32] and HMBD [43]) using Dereplicator+ [34]. Precursor and product mass tolerance of 0.002 Da were used. Since Dereplicator+ currently cannot identify molecules with smaller masses, in the case of molecular features with precursor masses below 200 Da, only parent mass matching is performed.

**Product identification.** For each identified substrate molecule, we used BioTransformer [29] to identify the product. Given a substrate molecule, Biotransformer predicts the molecular product of a biotransformation using a rule-based approach, where the rules are extracted through literature mining.

## Figures and Tables

**Table 1:** TransDiscovery identified 17 biotransformations. The columns $\rho_{s}$ and $\rho_{p}$ represent Spearsman’s rank correlation coefficients between the microbial features and substrate or product, respectively. The top biotransformations hold negative/positive biotransformations between substrates/products and microbial features, and the bottom ones do not.

Substrate Name	Biotransformation Name	List of All Strains that Are Observed	$\rho_{s}$	$\rho_{p}$	Description
Dihydroferuloylglycine	Hydrolysis of carboxylic acid ester	Prevotella, etc.	−0.16	0.17	Lachnospiraceae bacterium AM48-27BH
5-(3${}^{\prime }$,4${}^{\prime }$-dihydroxyphenyl)-valeric acid	Dehydroxylation	Pseudomonas; Enterobacteriaceae	−0.14	0.11	Escherichia coli DEC12C
Isoferulic acid; Ferulic acid	Alpha, beta-ketoalkene double bond reductase	Oscillospira, etc.	−0.11	0.15	Corynebacterium aurimucosum 911 CAUR
5-(3${}^{\prime }$-Methoxy-4${}^{\prime }$-hydroxyphenyl)-valerolactone	Dehydroxylation	Methanobrevibacter	−0.11	0.10	Methanobrevibacter woesei DSM 11979
3-Hydroxy-4-methoxyphenyllactic acid, etc.	Dehydroxylation	Dentocariosa	−0.11	0.09	Rothia dentocariosa 694 RDEN
Dihydrocaffeic acid	Catechol O-methylation	Tissierellaceae; Finegoldia	−0.10	0.16	Peptoniphilus senegalensis JC140
Hydroxybenzoic acid; Protocatechuic aldehyde	Dehydroxylation	Blautia	−0.09	0.09	Blautia wexlerae BIOML-A4
Dihydrosinapic acid, etc.	Dehydroxylation	Ruminococcaceae; Lachnospiraceae	−0.09	0.09	Lachnospiraceae bacterium MGYG-HGUT-00141
Matairesinol	Dehydroxylation	Prausnitzii	−0.08	0.11	Faecalibacterium prausnitzii MGYG-HGUT-00195
3-Phenylpropionic acid	Beta-Oxidation of carboxylic acid	Blautia	−0.08	0.09	Blautia wexlerae BIOML-A4
p-Coumaric acid; m-Coumaric acid	Dehydroxylase; Dehydroxylation	Faecalibacterium; Prausnitzii	0.13	0.14	Veillonella parvula BIOML-A2
Dihydroferulic acid	Dehydroxylation	Clostridiales; Granulicatella	0.11	0.14	Ruminococcus bromii ATCC 27255
3-Hydroxyphenylvaleric acid	Dehydroxylation	Enterobacteriaceae	0.11	0.11	Escherichia coli DEC12C
p-Coumaric acid	Decarboxylation of phenolic acid/hydroxycinnamic acid	Bifidobacterium; Clostridiales; Lactobacillus	0.10	0.17	Lactobacillus casei NBRC 101979
5-(3${}^{\prime }$,4${}^{\prime }$-dihydroxyphenyl)-valeric acid	Catechol O-methylation	Desulfovibrio; Enterobacteriaceae	0.10	−0.09	Ruminococcus torques 2789STDY5608867
Protocatechuic acid, etc.	Dehydroxylation; Aldehyde oxidation	Bacillales, etc.	−0.10	−0.10	Finegoldia magna DSM 20470
3-Hydroxyphenylpropionic acid; Paeonol, etc.	UDP-glucuronosyltransferase	Pseudomonas	−0.09	−0.09	Pseudomonas fragi F1786

If multiple strains are included in one row, the *ρ* value for the first strain is shown.

## Data Availability

The TransDiscovery computer code is available on GitHub at https://github.com/mohimanilab/TransDiscovery (accessed on 13 December 2021).

## Supplementary Materials

The following supplementary figures and tables are available online at https://www.mdpi.com/article/10.3390/metabo12020119/s1. Figure S1: Distribution of the samples counting molecular and microbial features that appear in the dataset. The label ’with-association’ represents features that form at least one significant correlation with *p*-value < $10^{-4}$ and abs(rho) > 0.1. None of the features present in less than 100 samples correspond to a significant correlation with other features. Features with more than 500 samples are not shown, Figure S2: The graph represents (a) 2364 substrate–product reaction pairs from BioTransformer between 370 phenol compounds and their predicted products. (b) A total of 45,479 molecule–molecule association pairs from the molecular network with 30,783 molecular features. (c) A total of 9,883,612 molecule–microbe association pairs from the association network between 30,783 molecular features and 11,265 microbial features. The numbers in the intercept area (d,e,f,g) represent numbers of pairs that exist in both (or all) studies, Figure S3: The graph represents (a) 200,833 substrate–product reaction pairs from BioTransformer between 41,919 HMDB molecules and their predicted products. (b) A total of 45,479 molecule–molecule association pairs from the molecular network between 30,783 molecular features. (c) A total of 9,883,612 molecule–microbe association pairs from the association network between 30,783 molecular features and 11,265 microbial features. The numbers in the intercept area (d,e,f,g) represent numbers of pairs that exist in both (or all) studies, Figure S4: The expected format for the input and output files in each step of TransDiscovery. The expected inputs for the association network are TSV files containing abundance tables for molecular and microbial features. The expected output is a TSV file with the statistic of the obtained associations. The expected input for molecular network is the mzML file containing the tandem mass spectrometry for each sample. The outputs are two TSV files with node information and edge information for the molecular network. The expected input for BioTransformer is an SMI file containing the smile strings for each molecule. The outputs are the reactions obtained by in silico search. The final output of TransDiscovery is a TSV file containing detailed information of every identified biotransformation, Figure S5: Integrating molecular and association networks. As the size of the network is enormous (41,765 nodes and 45,479 edges), here, we focus on some of the networking families that have a known molecule identified by Dereplicator as a polyphenol or a vitamin (25 molecules in total). As the association network is currently too dense to visualize, we only show the top two microbial features for each molecular feature (Fisher *p*-value of $1\times 10^{-5}$), Table S1: Mass annotation for matched molecular features obtained from AGP and molecules from PhenolDB, Table S2: Specific details of molecular features in identified biotransformations. The first two columns are the molecular masses for substrates and products. The following two columns are the *p*-values between the microbial features and substrate or product molecules, respectively. The last three columns are the number of samples that hold substrates, products, or substrate–product pairs for the 17 given biotransformations, Table S3: Identified biotransformations with enzymatic features as input. The enzymatic features were extracted from taxonomy-annotated microbial features using PICRUSt, Table S4: Biotransformations identified using enzymatic features from shotgun sequencing data with a *p*-value threshold of $10^{-3}$. Due to the limited sample size, none of these identified biotranformations are significant (with *p*-values less than $10^{-4}$), Table S5: Bacterial species annotation for identified strains. The scores listed in the table were obtained by matching the strain sequence with the NCBI RefSeq database using BLAST alignment.
