# Supplementary figures and images for: TransDiscovery: Discovering Biotransformation from Human Microbiota by Integrating Metagenomic and Metabolomic Data
Source: Metabolites. 2022 Jan 26;12(2):119. doi: 10.3390/metabo12020119 (PMC8877437; doi:10.3390/metabo12020119)

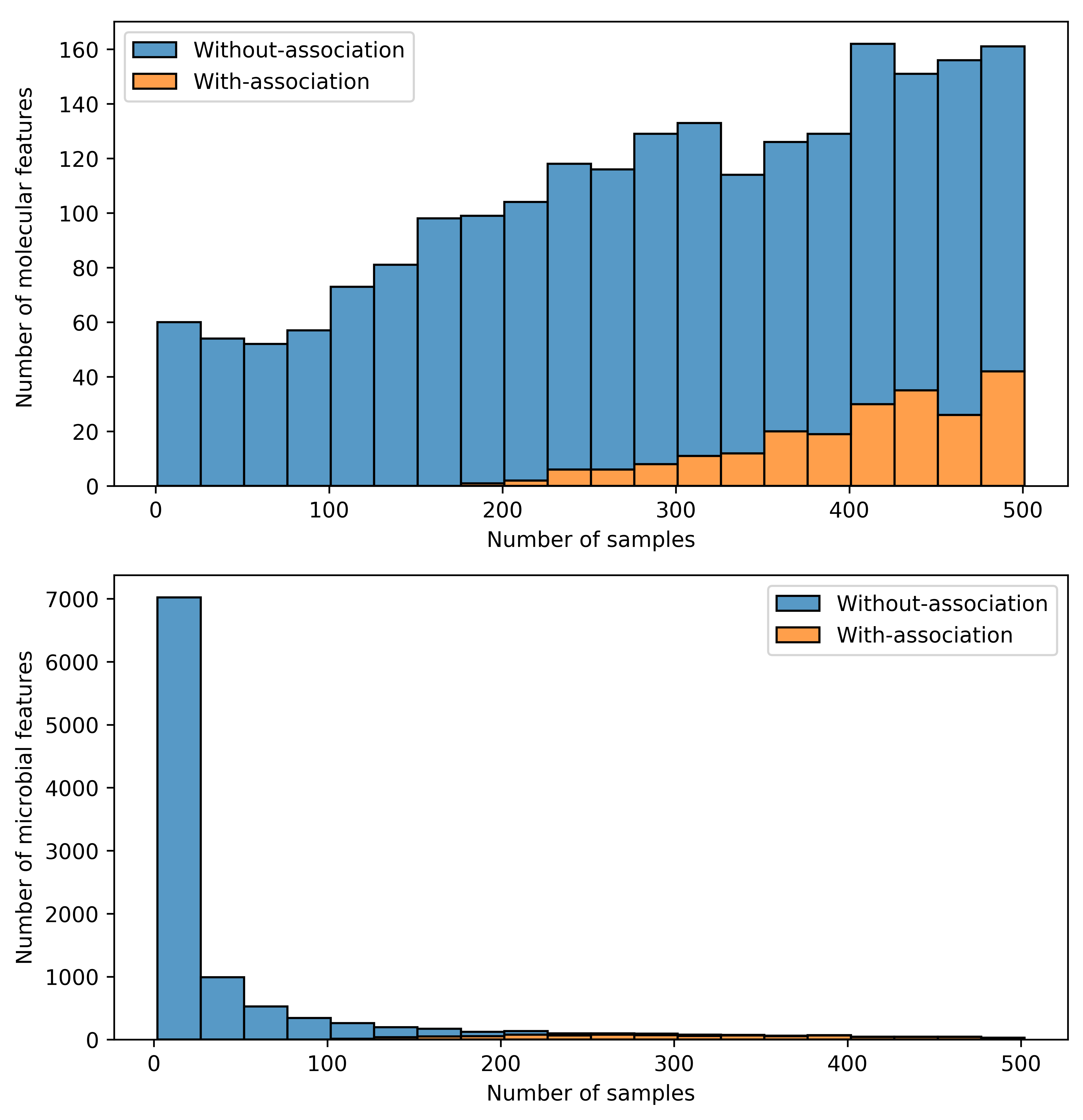

Supplement: Supplementary file 1 [file metabolites-12-00119-s001.zip › Supplementary_material_TransDiscovery/Fig S1.jpg]

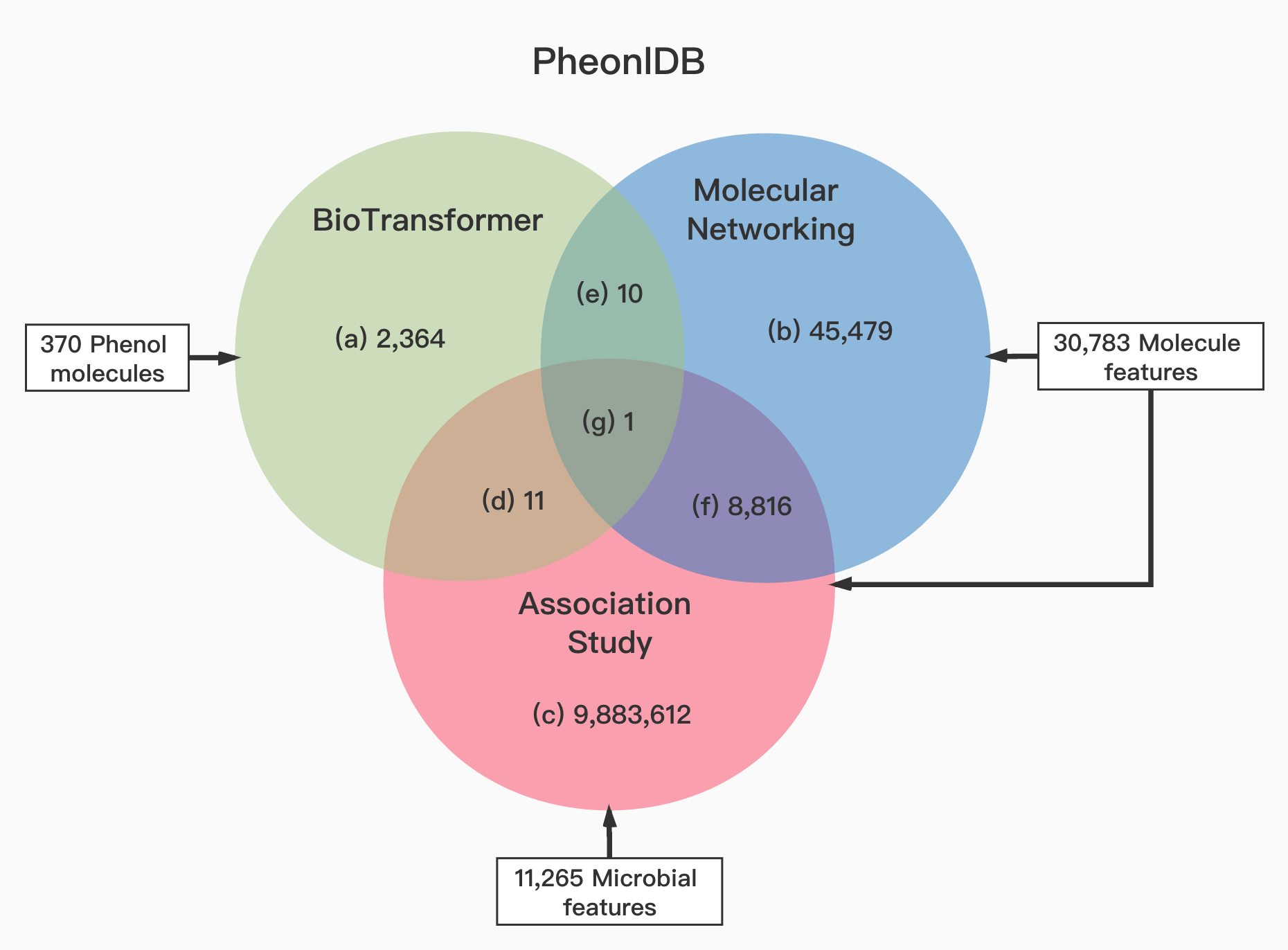

Supplement: Supplementary file 1 [file metabolites-12-00119-s001.zip › Supplementary_material_TransDiscovery/Fig S2.jpg]

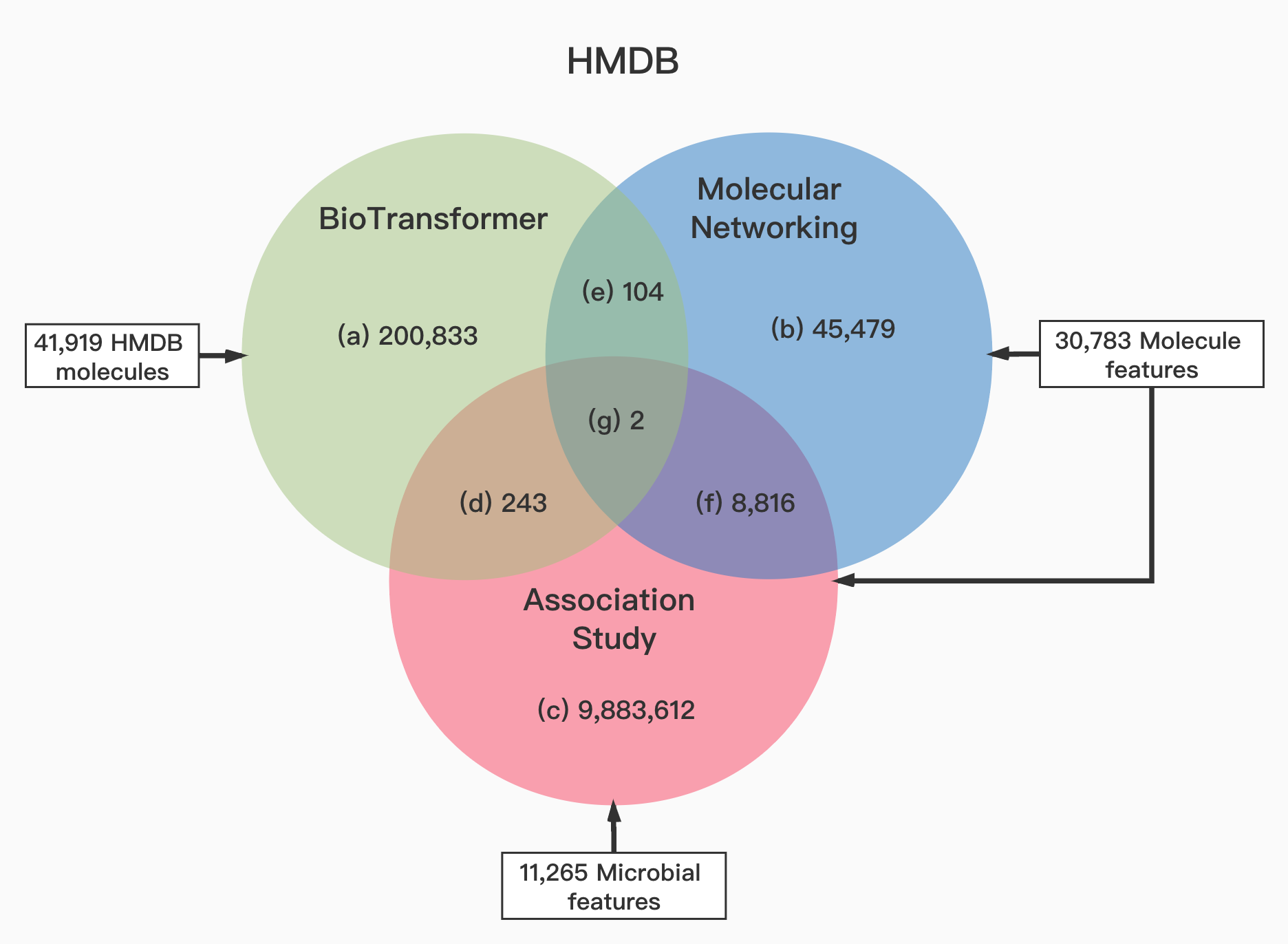

Supplement: Supplementary file 1 [file metabolites-12-00119-s001.zip › Supplementary_material_TransDiscovery/Fig S3.jpg]

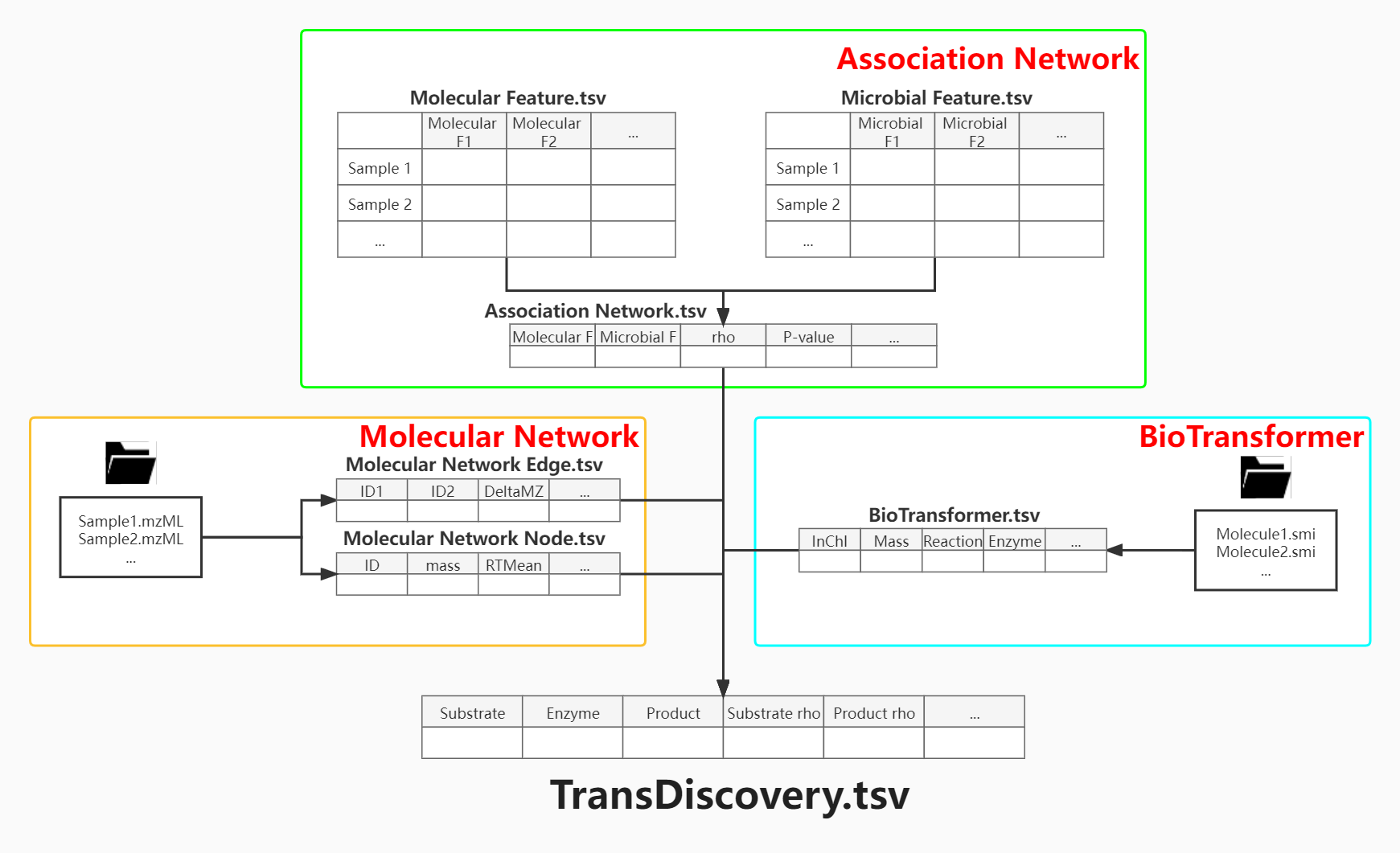

Supplement: Supplementary file 1 [file metabolites-12-00119-s001.zip › Supplementary_material_TransDiscovery/Fig S4.jpg]
